# Association of psychological symptoms with poor sleep quality in adults: a cross-sectional network analysis and effect modification by thyroid hormones

**DOI:** 10.3389/fendo.2026.1926090

**Published:** 2026-08-18

**Authors:** Mingxuan Xie, Qingrong Liu, Runxi Yang, Jiale He, Zhenghong Luo, Huixian Xiong, Ping Shuai, Xiaopeng Yao

**Affiliations:** 1 School of Public Health, Southwest Medical University, Luzhou, China; 2 Department of Health Management & Physical Examination, Sichuan Academy of Medical Sciences, Sichuan Provincial People’s Hospital (Affiliated Hospital of University of Electronic Science and Technology of China), Chengdu, China; 3 School of Medical Information and Engineering, Southwest Medical University, Luzhou, China; 4 Central Nervous System Product Research and Development Key Laboratory of Sichuan Province, Luzhou, China; 5 Medical Engineering & Medical Informatics Integration and Transformational Medicine of Luzhou Key Laboratory, Luzhou, China

**Keywords:** effect modification, free triiodothyronine, network analysis, poor sleep quality, psychological symptoms

## Abstract

**Introduction:**

Psychological symptoms and poor sleep quality (PSQ) frequently co-occur and impose a substantial public health burden. This study aimed to investigate the association between psychological symptoms and PSQ, examine whether thyroid hormones modify this association, and characterize the mixed psychological -sleep -thyroid symptom network among adults undergoing routine health examinations.

**Methods:**

This study enrolled 3,694 adults who underwent routine health screening at the Health Management Center of Sichuan Provincial People’s Hospital between April 2022 and December 2025. Participants were categorized into a PSQ group and a normal control group based on their Pittsburgh Sleep Quality Index (PSQI) scores. Modified Poisson regression with robust sandwich variance estimators (HC3) was used to estimate prevalence ratios (PRs) for the association between psychological symptoms (Symptom Checklist-90, SCL-90) and PSQ. Effect modification by thyroid-stimulating hormone (TSH), free triiodothyronine (FT3), and free thyroxine (FT4) was tested using multiplicative interaction terms in stratified and continuous-variable models. Finally, a mixed “psychological-sleep-thyroid” symptom network was constructed to calculate the expected influence and bridge strength of the nodes.

**Results:**

After adjusting for potential confounders, participants in the highest quartile of GSI scores exhibited a 5.81-fold increased prevalence of PSQ compared to those in the lowest quartile (PR = 5.81, 95% CI: 4.86-6.95, P < 0.001). Effect modification analysis revealed that FT3 significantly modified the association between psychological symptoms and PSQ (PR for interaction per 1-unit GSI increase = 1.35, 95% CI: 1.24-1.46, P < 0.001), with the prevalence ratio increasing from 1.61 (95% CI: 1.53-1.69) in the below-median FT3 group to 2.26 (95% CI: 2.02-2.53) in the above-median FT3 group. The modifying effects of TSH and FT4 were not statistically significant in continuous models. Symptom network analysis demonstrated that depressive symptoms, anxiety, and subjective sleep quality were among the nodes with the highest expected influence. Moreover, daytime dysfunction and FT3 demonstrated substantial cross-module bridge expected influence.

**Discussion:**

In this cross-sectional study, elevated psychological symptom severity was positively associated with PSQ prevalence, and this association appeared to be modified by FT3 levels. Depressive symptoms and subjective sleep quality emerged as central nodes, while daytime dysfunction and FT3 appeared to serve as key bridge nodes across psychological, sleep, and thyroid domains. Longitudinal studies are needed to clarify temporal relationships and evaluate whether targeting FT3-related pathways and daytime functioning may help reduce sleep disturbances.

## Graphical Abstract

**Figure:**
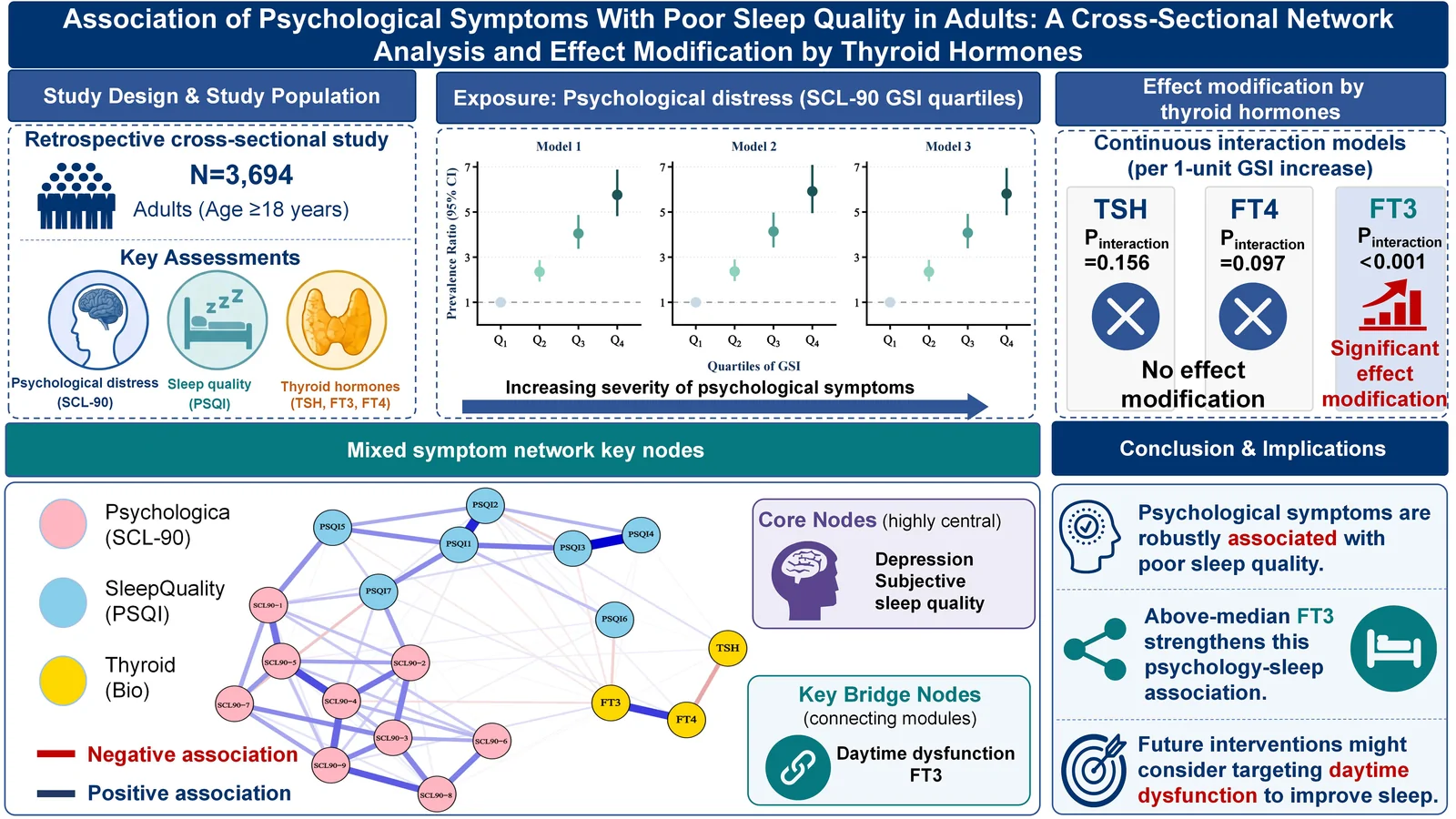

## 1 Introduction

Driven by the accelerating pace of modern life and intensifying societal competition, psychological symptoms and Poor sleep quality (PSQ) frequently co-occur as highly comorbid conditions, emerging as a prominent global public health challenge (1). The chronic persistence of these two conditions not only severely impairs individual cognitive function and diminishes quality of life, but also serves as an independent risk factor for cardiovascular diseases, metabolic disorders, and psychiatric illnesses, thereby imposing a profound burden on public health prevention and control systems (2, 3). To mitigate this burden, proactive screening is essential. Adults undergoing routine health examinations represent a critical target population, offering a valuable window to identify both psychological symptoms and PSQ before clinically overt diseases develop. Consequently, the early identification of PSQ and psychological symptoms in this population, along with the elucidation of their intrinsic associations and regulatory mechanisms, holds significant practical implications for mitigating subsequent disease burdens and enhancing the precision of population health management.

Existing research indicates a significant positive association between psychological symptoms and PSQ, wherein specific manifestations-such as depression, anxiety, and somatization—act as crucial associated factors for PSQ onset (4), and underlying cognitive processes may further mediate this relationship (5). However, the neuroendocrine biological mechanisms associated with both psychological states and sleep rhythms have yet to be systematically elucidated. As the core effector products of the hypothalamic-pituitary-thyroid (HPT) axis, thyroid hormones not only participate in the basal regulation of systemic metabolism, neurodevelopment, and sleep-wake rhythms (6), but their fluctuations are also closely linked to neuroendocrine dysregulation induced by chronic psychological stress, serving as an important correlate between psychological states and physiological functions (7).

To date, studies investigating thyroid hormones, psychological symptoms, and PSQ have predominantly relied on unidirectional association analyses. No study has yet to clarify whether thyroid hormones act as an effect modifier that alters the strength of the association between psychological symptoms and PSQ. Elucidating this modifying effect would facilitate the identification of high-prevalence subpopulations prone to PSQ when confronted with psychological stress, thereby providing an evidence base for precision health management. Furthermore, traditional epidemiological studies typically treat psychological symptoms, thyroid hormones, and PSQ as independent variables in linear analyses, which are inadequate for comprehensively capturing the interactive characteristics within the complex human biopsychological system (8). Symptom Network Analysis (SNA) offers a novel perspective for unraveling complex comorbidity mechanisms by quantifying and characterizing the network interactions and potential bridge nodes across multidimensional indicators (9), as demonstrated in recent comorbidity network studies (10).

Building on this premise, the present study aims to investigate the association between multidimensional psychological symptoms and sleep quality, and to examine the potential effect modification by thyroid-stimulating hormone (TSH), free triiodothyronine (FT3), and free thyroxine (FT4). Additionally, by employing symptom network analysis, this study seeks to construct a mixed “psychological-sleep-thyroid” symptom network to identify the core and potential bridge nodes within this system. This study aims to provide epidemiological evidence regarding the association among psychological symptoms, thyroid hormones, and poor sleep quality, while generating hypotheses concerning their potential neuroendocrine relationships.

## 2 Methods

### 2.1 Subjects of the study

This retrospective study enrolled individuals who underwent routine health examinations at the Health Management Center of Sichuan Provincial People’s Hospital between April 1, 2022, and December 31, 2025. The study population primarily comprised two categories: (1) individuals undergoing self-referred health check-ups; and (2) active and retired personnel participating in employer-sponsored health screenings across various sectors (e.g., government agencies, public institutions, education, and manufacturing, representing diverse occupational backgrounds).The inclusion criteria were as follows: (1) adult examinees (aged ≥ 18 years); and (2) completion of the Symptom Checklist-90 (SCL-90) and the Pittsburgh Sleep Quality Index (PSQI) assessments, alongside complete laboratory data for serum thyroid hormones (TSH, FT3, and FT4).Participants were excluded if they met any of the following criteria: (1) a documented history of hyperthyroidism, hypothyroidism, thyroiditis, or prior thyroid surgery/radioiodine therapy; (2) the presence of severe hepatic or renal dysfunction, acute infectious diseases, or autoimmune disorders; or (3) pregnancy or lactation. Initially, a total of 4,076 enrolled individuals who completed the SCL-90 assessment during the study period were screened. We subsequently excluded 382 participants based on the following criteria: 95 individuals with incomplete PSQI data, 239 individuals missing thyroid function indices, and 48 individuals diagnosed with hyperthyroidism. After these step-wise exclusions, a final analytical sample of 3,694 participants was included in the current study (see **Supplementary Figure 1** for the detailed flowchart). Given the retrospective design of this study, the research protocol was officially reviewed and approved by the Clinical Ethics Committee of Sichuan Provincial People’s Hospital in 2026 (Ethical Approval No.2026-378). Following this ethical approval, the retrospective extraction of completely de-identified historical data (from examinations originally conducted between April 2022 and December 2025) and subsequent statistical analyses were performed in 2026. Consequently, the requirement for informed consent was waived.

### 2.2 Questionnaire

#### 2.2.1 Sociodemographic and lifestyle variables

Basic demographic characteristics (e.g., age and sex), lifestyle factors, and medical history were collected utilizing a standardized hospital questionnaire. Alcohol consumption was defined as drinking alcohol at least once per week for ≥6 consecutive months (11). Smoking was defined as smoking ≥1 cigarette per day for ≥6 consecutive months (12).

#### 2.2.2 Pittsburgh sleep quality index

The PSQI, originally developed by Buysse et al (13), was administered using the Chinese version translated and validated by Liu et al. (14). This instrument evaluates seven dimensions: subjective sleep quality, sleep latency, sleep duration, habitual sleep efficiency, sleep disturbances, use of sleeping medications, and daytime dysfunction. Each dimension is scored on a scale from 0 to 3, yielding a global score ranging from 0 to 21.

#### 2.2.3 Symptom checklist-90

Psychological symptoms were evaluated using the 90-item SCL-90 inventory, which encompasses nine symptom dimensions (factors): somatization, obsessive-compulsive, interpersonal sensitivity, depression, anxiety, hostility, phobic anxiety, paranoid ideation, and psychoticism. Each item is rated on a 5-point Likert scale (ranging from 1 to 5), generating a total score between 90 and 450. In this study, the term ‘psychological symptoms’ is defined as the overarching clinical concept encompassing various multidimensional manifestations of mental health burden. The severity of these psychological symptoms was quantitatively measured using the Global Severity Index (GSI), a specific statistical metric calculated as the mean score of all 90 items (ranging from 1 to 5). Higher GSI scores indicate greater psychological symptom severity (15). To minimize construct overlap with the PSQI sleep components, the SCL-90 additional sleep-related items were excluded, and the GSI was calculated based on the remaining items.

#### 2.2.4 Quality assurance

To ensure data integrity and the clinical rigor of the assessments, all sleep and psychological questionnaires were administered within a specialized psychological outpatient setting. Certified psychological professionals distributed the scales, provided on-site guidance, and offered standardized clarifications for any ambiguous items during the data collection process, thereby minimizing the potential impact of cognitive biases on the results.

### 2.3 Physical examinations

Blood pressure was measured by trained healthcare professionals following standardized procedures using a fully automated electronic sphygmomanometer (HBP-9020, Omron). Measurements were taken while participants were in a resting seated position. Three consecutive readings were obtained at 2-minute intervals, and the average value was recorded for analysis. Height and weight were measured using an ultrasonic stadiometer and scale (HNH-318, Omron), with participants standing barefoot and wearing light clothing. Body mass index (BMI) was subsequently calculated. Waist circumference (WC) was measured using a non-stretchable measuring tape at the midpoint between the lower rib margin and the iliac crest.

### 2.4 Biochemical assessments

Fasting venous blood samples (4–5 mL) were drawn from all participants in the morning (between 07:30 and 10:30). These samples were subsequently analyzed at the Clinical Laboratory Center of Sichuan Provincial People’s Hospital to determine the following biochemical parameters: fasting blood glucose (FPG), total cholesterol (TC), triglycerides (TG), high-density lipoprotein cholesterol (HDL-C), low-density lipoprotein cholesterol (LDL-C), and thyroid function markers, including TSH, FT4, and FT3.

In this study, TSH, FT4, and FT3 were selected to evaluate thyroid function, as they collectively reflect the regulatory dynamics of the HPT axis. Specifically, TSH acts as the most sensitive indicator of pituitary-thyroid feedback; FT4 is the predominant secretory product of the thyroid gland; and FT3 represents the biologically active form. Collectively, these three indices provide a comprehensive assessment of thyroid hormone synthesis, secretion, and peripheral conversion.

### 2.5 Definitions

#### 2.5.1 Poor sleep quality

In accordance with widely adopted clinical epidemiological standards in China, PSQ was defined using a PSQI global score cut-off of > 7. Although polysomnography (PSG) is considered the clinical gold standard for evaluating sleep disorders, the PSQI has been extensively validated for large-scale epidemiological studies because of its favorable validity, reliability, and feasibility (16, 17). Consequently, participants were stratified into the PSQ group (score > 7) or the normal control group (score ≤ 7, indicative of normal sleep quality) (18). To ensure the robustness of our findings, a sensitivity analysis was subsequently performed utilizing the internationally established cut-off threshold of a PSQI score > 5 (19).

#### 2.5.2 Hypertension

Following the Chinese Guidelines for the Prevention and Treatment of Hypertension, hypertension was defined as meeting any of the following criteria: a systolic blood pressure (SBP) ≥ 140 mmHg, a diastolic blood pressure (DBP) ≥ 90 mmHg (1 mmHg = 0.133 kPa), a documented clinical history of hypertension, or the current use of antihypertensive medications (20).

#### 2.5.3 Diabetes mellitus

Based on the Chinese Guidelines for the Prevention and Treatment of Type 2 Diabetes, diabetes was diagnosed if participants presented with a FPG ≥ 7.0 mmol/L, a glycosylated hemoglobin (HbA1c) level ≥ 6.5%, or a prior clinical diagnosis of diabetes (21).

### 2.6 Statistical analysis

All statistical analyses were conducted using SPSS software (version 27.0) and R software (version 4.4.3). Continuous variables were expressed as the mean ± standard deviation (SD) if normally distributed, and between-group comparisons were performed using the independent samples t-test. Non-normally distributed continuous variables were summarized as the median with interquartile range (IQR) and analyzed using the Wilcoxon rank-sum test (Mann-Whitney U test). Categorical variables were presented as frequencies and percentages (%), with differences evaluated via Pearson’s Chi-square test. Given the retrospective design, no formal *a priori* power calculation was conducted. The final sample of 3,694 participants (1,485 PSQ cases) far exceeded the minimum of approximately 280 outcome events required for the fully adjusted model containing 14 parameters (10–20 events per predictor), ensuring stable estimates.

Given the relatively high prevalence of PSQ, modified Poisson regression models with robust sandwich variance estimators were used to estimate PRs and 95%CIs for the association between psychological symptom severity and PSQ. Psychological symptom severity was analyzed both as quartiles of the GSI and as a continuous variable, and linear trends across quartiles were assessed by modeling the median value of each quartile as a continuous variable. Multivariable models were adjusted for age, sex, body mass index (BMI), smoking status, alcohol consumption, systolic blood pressure (SBP), fasting plasma glucose (FPG), total cholesterol (TC), hypertension, and diabetes. Variance inflation factors (VIFs) were calculated to assess multicollinearity.

Effect modification by thyroid hormones (TSH, FT3, and FT4) was evaluated by including multiplicative interaction terms in the modified Poisson regression models. Interaction analyses were performed using both dichotomized (median split) and continuous thyroid hormone variables. Sensitivity analyses were conducted by restricting the analyses to participants with thyroid hormone concentrations within the laboratory reference ranges.

#### 2.6.1 Network analysis

To properly accommodate the diverse measurement levels and non-normal distributions of our variables, a mixed symptom network was estimated using the Mixed Graphical Models (MGM) framework via the mgm package in R (version 4.4.3) (22). The network comprised 19 nodes: nine SCL-90 dimensions (psychological module), seven PSQI components (sleep module), and three individual thyroid hormones (thyroid module; retained individually to map their specific proximities). Modules were defined *a priori* based on theoretical domains. During model estimation, L_1_-regularization (LASSO) coupled with the Extended Bayesian Information Criterion (EBIC) was applied. This regularization approach naturally handles potential multicollinearity among physiological thyroid indices and shrinks spurious connections to zero. Notably, a severe floor effect was observed for PSQI component 6 (sleeping medication use), with 91.8% of the participants scoring 0. This node was retained to preserve the complete PSQI construct; the inherent LASSO penalty effectively prevents such low-variance nodes from distorting the overall network structure. Centrality was quantified using Expected Influence (EI), and cross-module connectivity was assessed using Bridge Expected Influence (Bridge EI). Network accuracy was evaluated via non-parametric bootstrapping (2,000 iterations), and stability was rigorously assessed using case-dropping correlation stability (CS) coefficients (23).

## 3 Results

### 3.1 Comparison of characteristics between the PSQ and normal control groups

**Figure 1:**
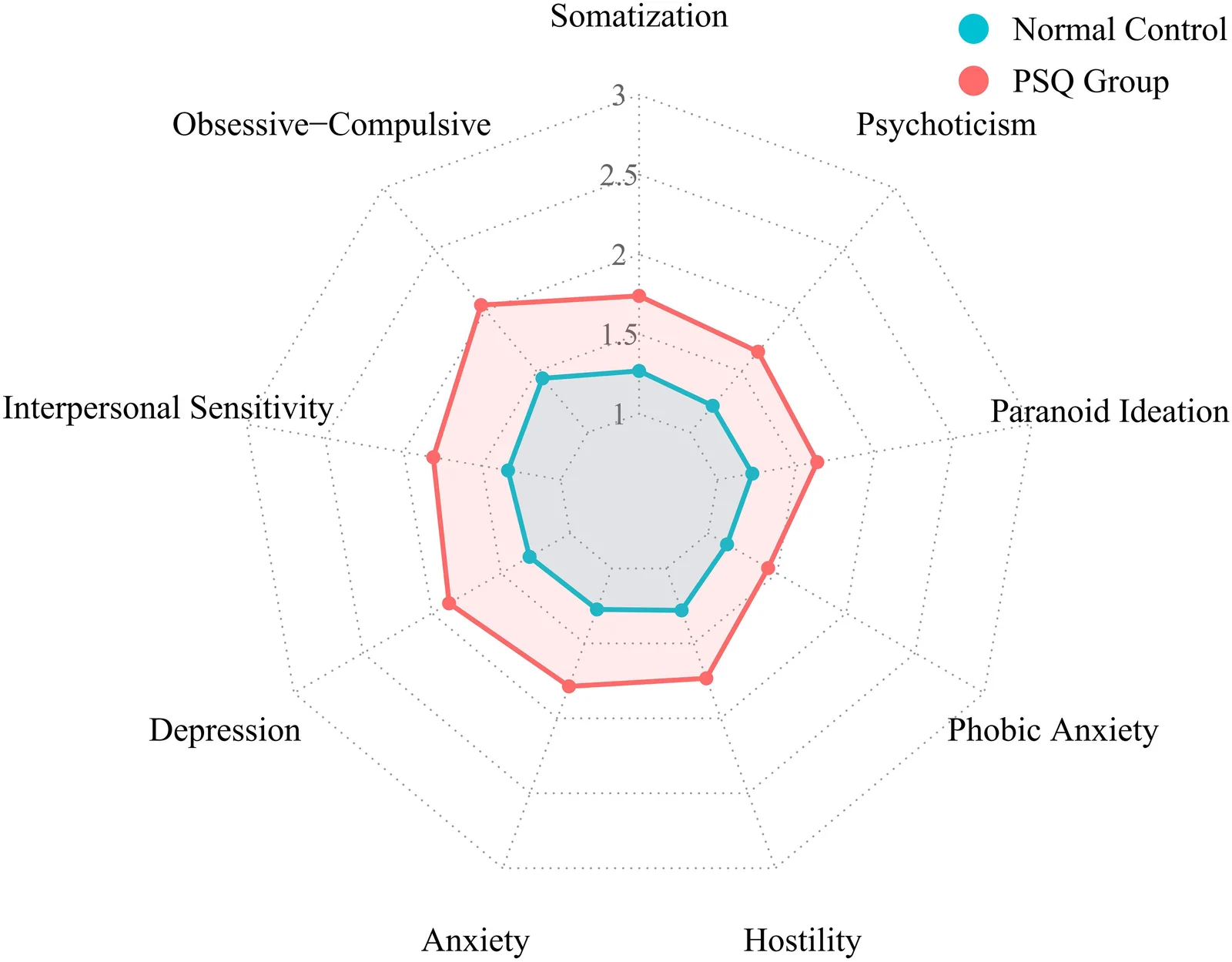
Radar chart comparing SCL-90 factor scores between the PSQ and normal control groups.

A total of 3,694 participants were included in this study and stratified into the PSQ group and the normal control group based on their PSQI scores. The overall cohort comprised 2,100 males (56.85%) and 1,594 females (43.15%), with a mean age of 38.61 ± 11.19 years. Between-group comparisons revealed that TSH levels were significantly elevated in the PSQ group compared to the normal control group, whereas FT3 levels were significantly lower (both P < 0.001). Additionally, the PSQ group exhibited significantly higher proportions of females, smokers, and alcohol drinkers than the control group (all P < 0.05). No statistically significant differences were observed between the two cohorts regarding FT4 levels, history of diabetes or hypertension, BMI, WC, FPG, blood pressure, or lipid profiles (including TG, TC, LDL-C, and HDL-C) (all P > 0.05), see **Table 1**. Regarding psychological symptom profiles, participants with PSQ consistently exhibited higher scores across all nine SCL-90 dimensions than those in the normal control group (**Figure 1**).

**Table 1:** Comparison of characteristics between the PSQ and normal control groups.

Characteristics	Total (n = 3694)	Normal group (n = 2209)	PSQ group (n = 1485)	Statistic	*P*
Age(years)	38.61 ± 11.19	39.06 ± 11.22	37.95 ± 11.12	t=2.95	**0.003**
TSH (mIU/L)	2.87 ± 1.68	2.68 ± 1.63	3.16 ± 1.72	t=-8.65	**<.001**
FT3(pmol/L)	4.91 ± 0.71	5.08 ± 0.64	4.68 ± 0.73	t=17.13	**<.001**
FT4(pmol/L)	16.67 ± 2.21	16.72 ± 2.19	16.59 ± 2.25	t=1.73	0.084
TC(mmol/L)	4.71 ± 0.92	4.71 ± 0.92	4.72 ± 0.91	t=-0.24	0.807
LDL-C(mmol/L)	2.83 ± 0.78	2.84 ± 0.79	2.82 ± 0.78	t=0.61	0.544
HDL-C(mmol/L)	1.38 ± 0.37	1.38 ± 0.37	1.39 ± 0.37	t=-0.73	0.463
FPG (mmol/L)	4.78 ± 1.01	4.80 ± 1.05	4.75 ± 0.95	t=1.56	0.118
BMI (kg/m^2^)	23.71 ± 3.68	23.69 ± 3.60	23.73 ± 3.80	t=-0.29	0.770
SBP (mmHg)	115.59 ± 15.36	115.55 ± 15.27	115.66 ± 15.49	t=-0.22	0.823
DBP (mmHg)	71.36 ± 10.95	71.23 ± 10.86	71.55 ± 11.08	t=-0.87	0.384
WC (cm)	79.57 ± 10.98	79.59 ± 10.68	79.56 ± 11.40	t=0.08	0.934
TG(mmol/L)	1.23 (0.84, 1.90)	1.24 (0.84, 1.90)	1.23 (0.83, 1.88)	Z=-0.30	0.765
GSI	1.31 (1.11, 1.67)	1.17 (1.07, 1.40)	1.60 (1.33, 2.10)	Z=-30.43	**<.001**
Sex, n (%)				χ²=4.19	**0.041**
Male	2100 (56.85)	1286 (58.22)	814 (54.81)		
Female	1594 (43.15)	923 (41.78)	671 (45.19)		
Smoking status, n (%)				χ²=7.31	**0.007**
No	2939 (79.56)	1790 (81.03)	1149 (77.37)		
Yes	755 (20.44)	419 (18.97)	336 (22.63)		
Alcohol drinker, n (%)				χ²=13.59	**<.001**
No	3098 (83.87)	1893 (85.69)	1205 (81.14)		
Yes	596 (16.13)	316 (14.31)	280 (18.86)		
Type 2 diabetes, n (%)				χ²=0.02	0.886
No	3574 (96.75)	2138 (96.79)	1436 (96.70)		
Yes	120 (3.25)	71 (3.21)	49 (3.30)		
Hypertension, n (%)				χ²=1.36	0.244
No	3293 (89.14)	1980 (89.63)	1313 (88.42)		
Yes	401 (10.86)	229 (10.37)	172 (11.58)		
Quartiles of GSI				χ²=861.87	**<.001**
* Q*_1_	889 (24.07)	800 (36.22)	89 (5.99)		
* Q*_2_	935 (25.31)	690 (31.24)	245 (16.50)		
* Q*_3_	929 (25.15)	460 (20.82)	469 (31.58)		
* Q*_4_	941 (25.47)	259 (11.72)	682 (45.93)		

Bold values indicate statistically significant differences with P < 0.05.

### 3.2 Association of psychological symptom severity with the prevalence of poor sleep quality

Poisson regression models were utilized to examine the association between psychological symptoms and the prevalence of PSQ. The results demonstrated a significant positive association between the continuous SCL-90 global score and the prevalence of PSQ. Further analysis stratifying participants into quartiles based on their GSI scores revealed a significant dose-response relationship. Compared to the lowest quartile (*Q*_1_), participants in the *Q*_2_, *Q*_3_, and *Q*4 groups exhibited a significantly increased prevalence of PSQ (all P < 0.001), with the prevalence increasing progressively as sleep-excluded GSI scores increased (P for trend < 0.001). This positive association remained robust even after adjusting for potential confounders; the adjusted PRs and 95% CIs for the *Q*_2_, *Q*_3_, and *Q*_4_ groups were 2.35 (1.93–2.88), 4.08 (3.39–4.92), and 5.81 (4.86–6.95), respectively (all P < 0.001), see **Table 2**. Two sensitivity analyses confirmed the robustness of these findings (**Supplementary Table 1**). The association remained consistent when using the PSQI > 5 cut-off (*Q*_4_ PR = 2.94, 95% CI: 2.66–3.24, P for trend < 0.001) and after excluding 675 participants with subclinical thyroid dysfunction (euthyroid *Q*_4_ PR = 5.81, 95% CI: 4.75–7.10, P for trend < 0.001). Additionally, all nine SCL-90 dimensions were independently associated with PSQ (**Supplementary Table 3**), particularly somatization (PR = 1.93), obsessive-compulsive symptoms (PR = 1.81), and psychoticism (PR = 1.79). Finally, multicollinearity among covariates was negligible (all VIFs < 2.0, **Supplementary Table 2**).

**Table 2:** Poisson regression analysis of the association between GSI quartiles and PSQ.

Variables	Model 1		Model 2		Model 3	
	*PR* (95%*CI*)	*P*	*PR* (95%*CI*)	*P*	*PR* (95%*CI*)	*P*
GSI (continuous)	1.93 (1.84, 2.02)	**<0.001**	1.93 (1.84, 2.03)	**<0.001**	1.91 (1.82, 2.01)	**<0.001**
Quartiles of GSI						
* Q*_1_	1.00 (Reference)		1.00 (Reference)		1.00 (Reference)	
* Q*_2_	2.35 (1.92, 2.87)	**<0.001**	2.37 (1.94, 2.90)	**<0.001**	2.35 (1.93, 2.88)	**<0.001**
* Q*_3_	4.05 (3.37, 4.87)	**<0.001**	4.14 (3.43, 4.98)	**<0.001**	4.08 (3.39, 4.92)	**<0.001**
* Q*_4_	5.76 (4.82, 6.88)	**<0.001**	5.92 (4.95, 7.09)	**<0.001**	5.81 (4.86, 6.95)	**<0.001**
*P* for trend	**<0.001**		**<0.001**		**<0.001**	

Model 1: unadjusted; Model 2: adjusted for age, sex; Model 3: further adjusted for smoking, alcohol drinking, diabetes, hypertension. Bold values indicate statistically significant differences with P < 0.05.

### 3.3 Effect modification by thyroid hormones on the association between psychological symptoms and PSQ

**Figure 2:**
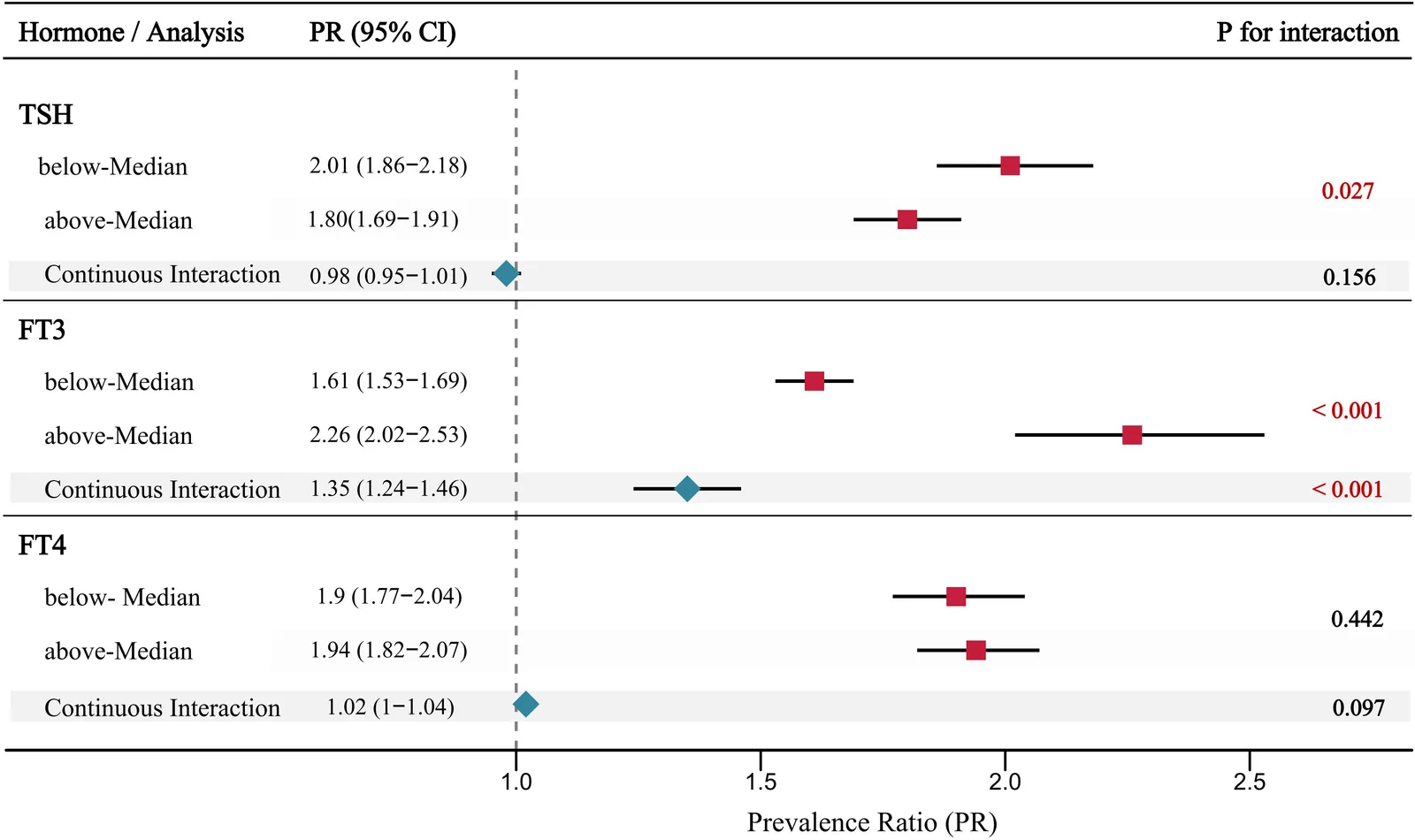
Effect modification by thyroid hormone levels on the association between psychological symptoms and poor sleep quality.

FT3 significantly modified the association between psychological symptoms and PSQ in both median-stratified (PR: 1.61 [95% CI: 1.53-1.69] vs. 2.26 [95% CI: 2.02-2.53]) and continuous interaction models (PR interaction = 1.35, 95% CI: 1.24-1.46, P < 0.001), with full adjustment for Model 3 covariates (**Figure 2**). Neither FT4 (median-stratified P = 0.442; continuous PR for interaction= 1.02, 95% CI: 1.00-1.04, P = 0.097) nor TSH (median-stratified P = 0.027; continuous PR for interaction = 0.98, 95% CI: 0.95-1.01, P = 0.156) showed consistent, robust modifying effects; the apparent TSH interaction in the stratified model was not replicated continuously and is interpreted as a chance finding. Restricted cubic spline analysis indicated significant nonlinearity in the FT3 main effect (P = 0.001) but not in the modifying effect (P = 0.194; **Supplementary Figure 2**). Additive interaction measures were negative (RERI = −0.28; AP = -0.21; SI = 0.57), reflecting a mathematical artifact of the inverse FT3 main effect. In the strictly euthyroid sub-cohort (n = 3,019, after excluding 675 participants with subclinical dysfunction), the FT3 interaction remained robust (PR for interaction = 1.44, 95% CI: 1.31-1.57, P < 0.001), as did the main effect (*Q*_4_ vs. *Q*_1_: PR = 5.81, 95% CI: 4.75-7.10). Sex-stratified analyses confirmed consistency across sexes (P for three-way interaction = 0.983), See **Supplementary Table 4****, 5**.

### 3.4 Stratified correlation patterns by FT3 level

**Figure 3:**
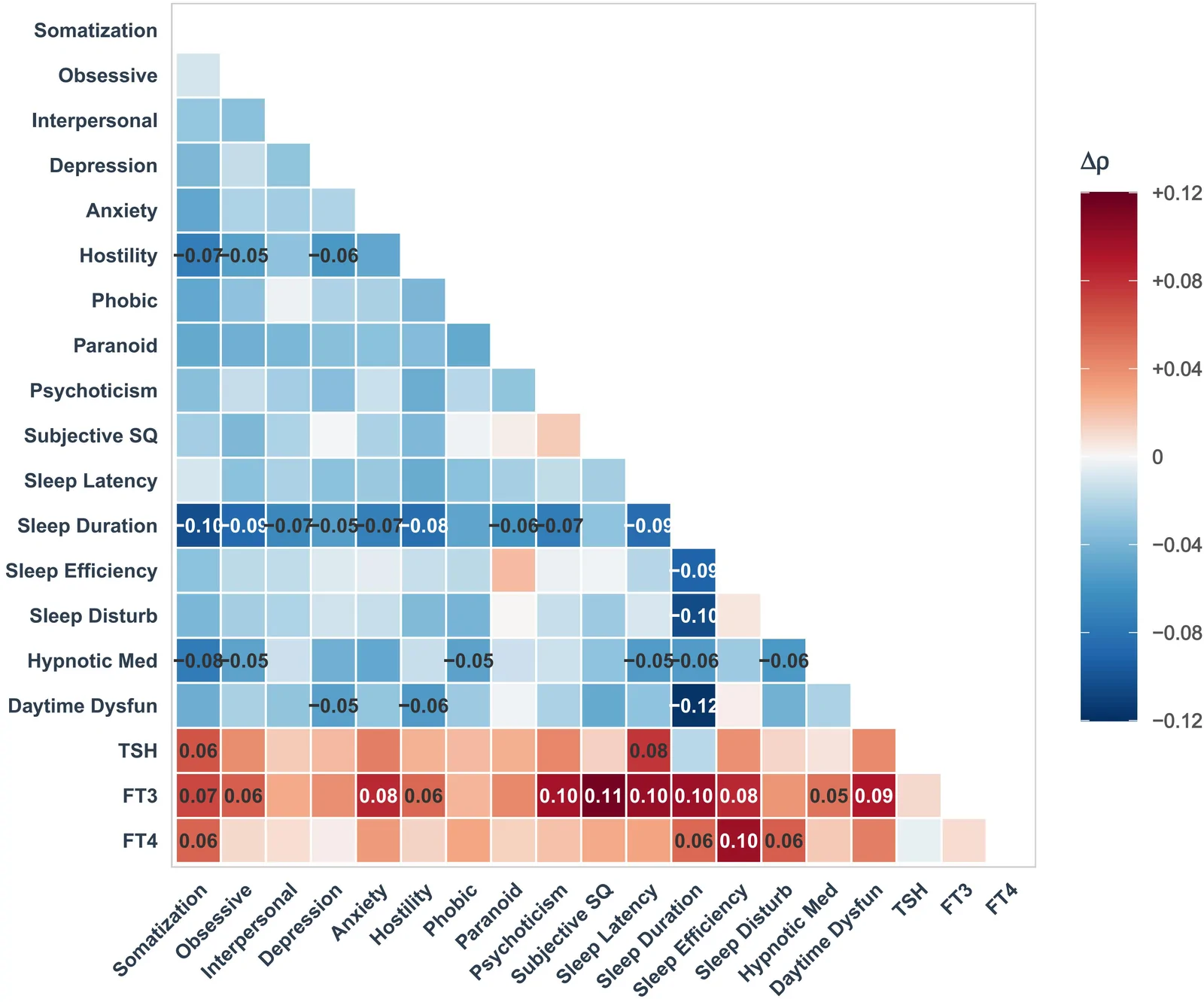
Effect modification by FT3 on inter-correlations among psychological symptoms, sleep quality, and thyroid hormones. Difference matrix (above-median FT3 − below-median FT3) showing divergence in Spearman correlation coefficients. Red indicates stronger correlations in the above-median FT3 group; blue indicates stronger correlations in the below-median FT3 group. Only |Δρ| > 0.05 annotated.

To visualize the effect modification by FT3, stratified Spearman correlation heatmaps were constructed by median FT3 level (**Supplementary Figure 3**). The difference matrix (**Figure 3**) revealed distinct correlation patterns between groups. In the above-median group, FT3 exhibited strengthened correlations with all PSQI components (Δρ = +0.05 to +0.11), whereas direct correlations between psychological symptoms and sleep parameters were attenuated (Δρ = −0.05 to −0.12). These findings indicate that the interconnection architecture of the psychological-sleep-thyroid system exhibits distinct correlational patterns across different FT3 strata. Specifically, the associations between psychological symptoms and sleep parameters appear attenuated, while the correlations involving FT3 are strengthened in the above-median FT3 group.

### 3.5 Construction of the mixed symptom network

**Figure 4:**
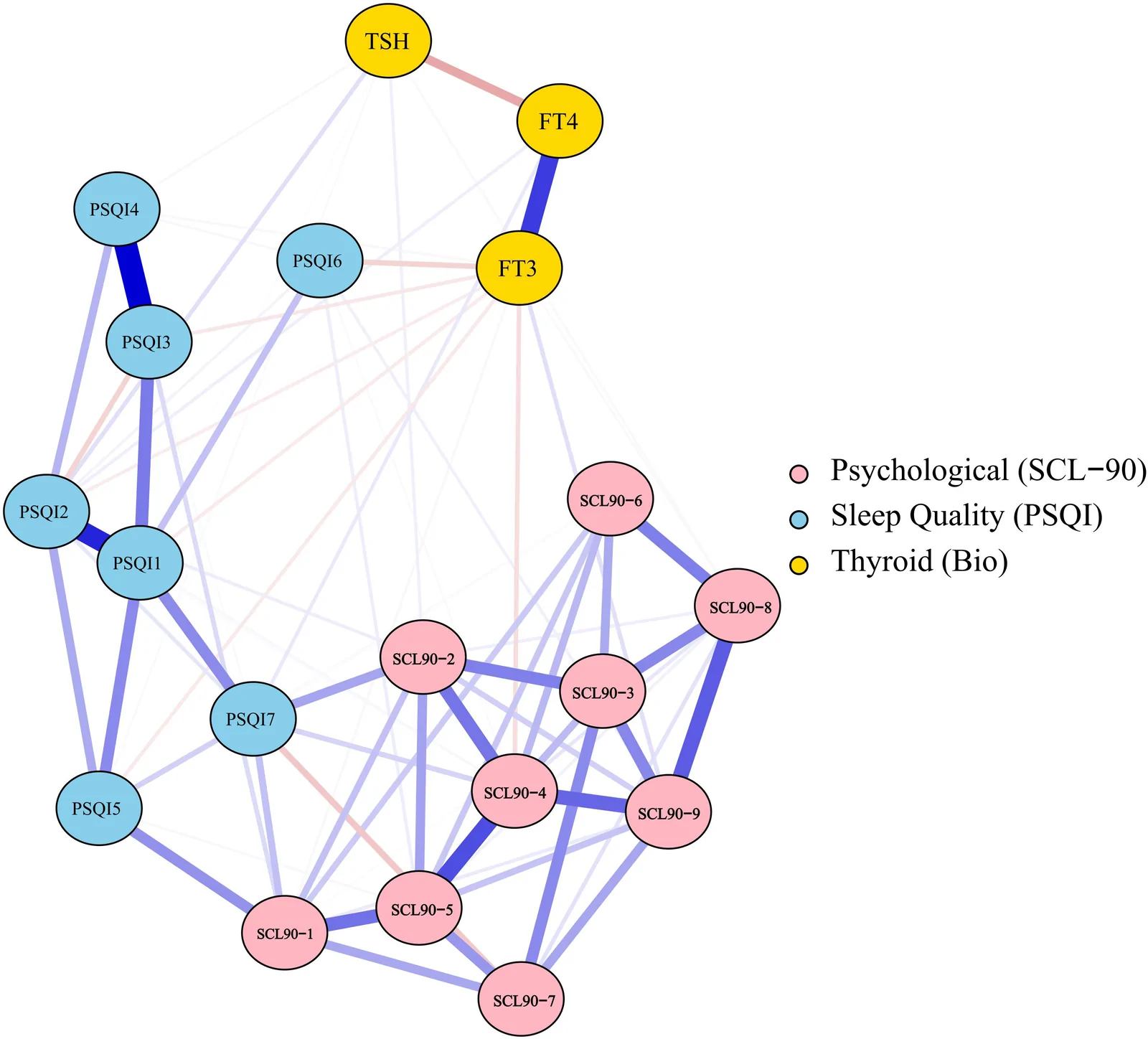
Mixed symptom network of psychological symptoms, sleep quality, and thyroid hormones. PSQI1: Subjective sleep quality; PSQI2: Sleep latency; PSQI3: Sleep duration; PSQI4: Habitual sleep efficiency; PSQI5: Sleep disturbances; PSQI6: Use of sleeping medications; PSQI7: Daytime dysfunction. SCL90-1: Somatization; SCL90-2: Obsessive-compulsive; SCL90-3: Interpersonal sensitivity; SCL90-4: Depression; SCL90-5: Anxiety; SCL90-6: Hostility; SCL90-7: Phobic anxiety; SCL90-8: Paranoid ideation; SCL90-9: Psychoticism. TSH: Thyroid-stimulating hormone; FT3: Free triiodothyronine; FT4: Free thyroxine.

The mixed symptom network integrating the SCL-90 dimensions, PSQI components, and thyroid hormone indices is illustrated in **Figure 4**. Within this network, the nodes represent individual items (variables). The edges (connecting lines) depict the partial correlation coefficients between these nodes, with the thickness of the edges proportional to the magnitude of the correlation; thus, thicker edges indicate stronger associations between variables. Furthermore, blue edges denote positive correlations, whereas red edges signify negative correlations.

### 3.6 Centrality and bridge indices of the integrated network

**Figure 5:**
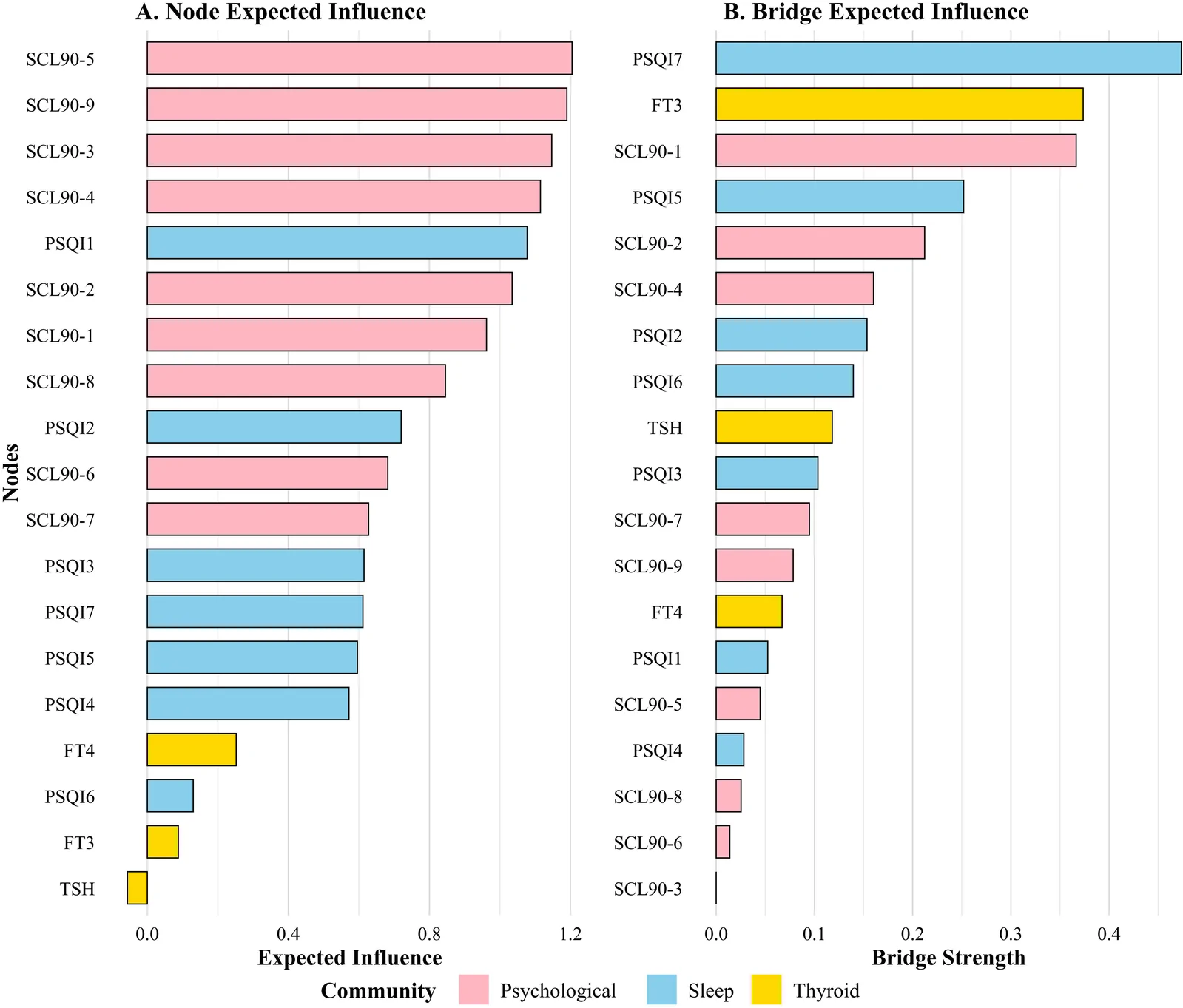
Centrality and bridge indices of the mixed symptom network. **(A)** Expected Influence (EI) of the nodes within the network. **(B)** Bridge Expected Influence (Bridge EI) of the nodes across the psychological, sleep, and thyroid modules. PSQI1: Subjective sleep quality; PSQI2: Sleep latency; PSQI3: Sleep duration; PSQI4: Habitual sleep efficiency; PSQI5: Sleep disturbances; PSQI6: Use of sleeping medications; PSQI7: Daytime dysfunction. SCL90-1: Somatization; SCL90-2: Obsessive-compulsive; SCL90-3: Interpersonal sensitivity; SCL90-4: Depression; SCL90-5: Anxiety; SCL90-6: Hostility; SCL90-7: Phobic anxiety; SCL90-8: Paranoid ideation; SCL90-9: Psychoticism. TSH: Thyroid-stimulating hormone; FT3: Free triiodothyronine; FT4: Free thyroxine.

Centrality analyses demonstrated that negative psychological symptoms and core sleep parameters were highly central within the network. Specifically, anxiety (EI = 1.20), psychoticism (EI = 1.19), interpersonal sensitivity (EI = 1.15), depression (EI = 1.11), and subjective sleep quality (EI = 1.08) exhibited among the highest Expected Influence (EI). Within the thyroid function module, FT3 demonstrated a higher expected influence (EI = 0.09) compared to both FT4 and TSH (**Figure 5A**). Bridge effect analysis further elucidated the cross-module interactive dynamics. “Daytime dysfunction” emerged as the node with the highest Bridge Expected Influence (Bridge EI = 0.47), serving as the primary hub connecting the sleep, psychological, and thyroid function modules. Notably, FT3 also displayed a critical bridge effect (Bridge EI = 0.37), a value substantially higher than those of TSH and FT4. Furthermore, “somatization” functioned as a key secondary bridge node (Bridge EI = 0.37) (**Figure 5B**). Detailed numerical values for all nodes are provided in **Supplementary Table 6**.

### 3.7 Accuracy and stability assessment of the mixed symptom network

**Figure 6:**
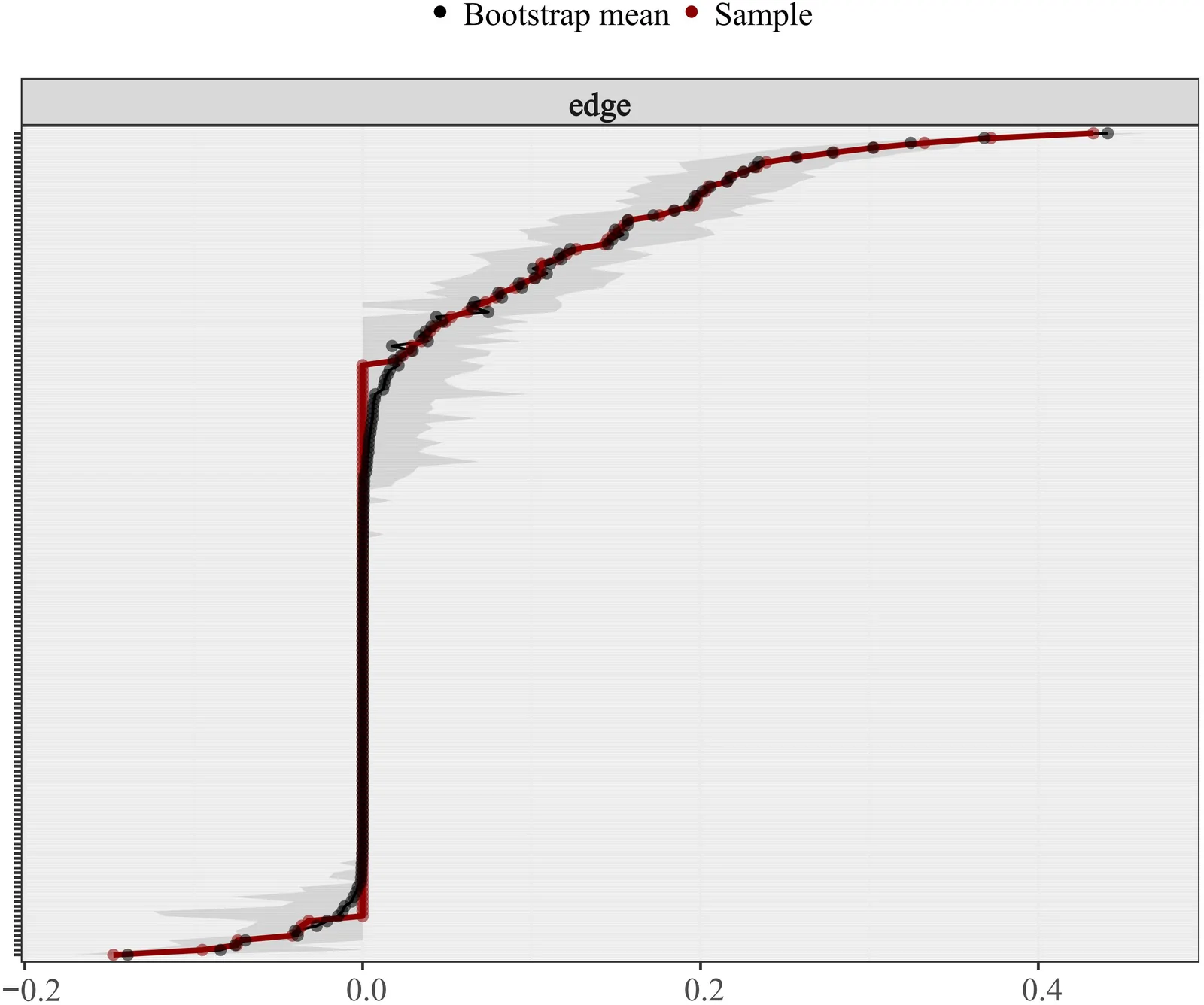
Accuracy assessment of network edge weights.

**Figure 7:**
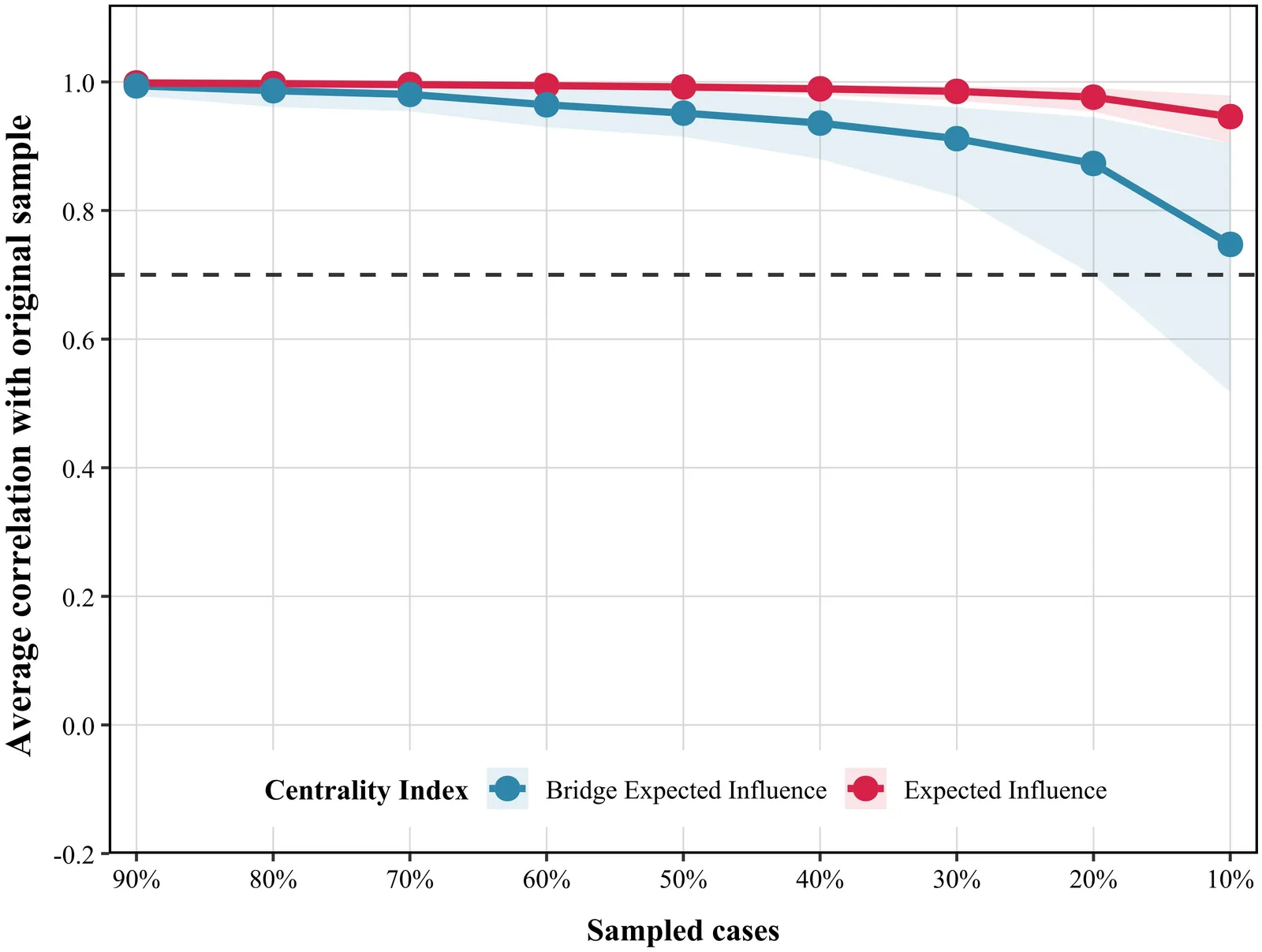
Stability assessment of centrality and bridge indices.

To evaluate the robustness of the constructed mixed symptom network, we employed a non-parametric bootstrapping approach (2000 iterations) to assess the accuracy of edge weights and the stability of centrality indices. The bootstrap analysis revealed that the 95%CIs for the edge weights were consistently narrow, and the original estimates were highly congruent with the bootstrap means, indicating high accuracy in edge weight estimation (**Figure 6**). Furthermore, the stability of the network indices was quantified using the CS coefficient. The CS coefficients for both Expected Influence and Bridge Expected Influence reached 0.9, substantially exceeding the recommended stringent threshold of 0.50. As visually verified by the case-dropping bootstrap curves (**Figure 7**), the average correlations with the original sample remained robustly high even when a substantial proportion of the sample was dropped, reflecting the immense statistical power provided by our large sample size. This indicates that the relative importance ranking of core nodes and cross-module bridge hubs remained highly consistent, even when a substantial proportion of the sample was removed. These findings collectively demonstrate the high robustness and reliability of the constructed network.

## 4 Discussion

This study examined the association between psychological symptoms and PSQ in health examination attendees and tested whether thyroid hormones modify this association. We found a dose-response relationship between psychological symptom severity and PSQ prevalence. Even after adjusting for potential confounders, this association remained robust, with participants in the highest quartile exhibiting a 5.81-fold increased prevalence of PSQ compared to those in the lowest quartile. To verify the robustness of these findings, sensitivity analyses were conducted using the internationally recognized cut-off point of PSQI > 5, which consistently supported our initial observations.

These results are consistent with previous epidemiological findings (24, 25), further confirming that psychological stressors, such as depression, anxiety, and somatization, are important associated factors for the onset of PSQ. While the link between PSQ and psychological symptoms has been extensively documented, this study offers an additional perspective by integrating multidimensional psychological symptoms, sleep quality, and objective thyroid hormone markers into a unified analytical framework. By employing effect modification analysis and SNA, we were able to quantify the complex interactive characteristics across these domains and identify pivotal bridge nodes spanning the psychological, endocrine, and sleep systems. These findings provide a theoretical and practical basis for clinical interventions, offering a more precise framework for navigating the intricate comorbidity networks in health management. It is noteworthy that the prevalence of PSQ in our cohort reached 40.2%, which is higher than the pooled estimates typically reported in general-population meta-analyses in China (18). This discrepancy can largely be attributed to the specific demographic characteristics of our study population and the assessment context. First, regarding the occupational and age profile, our sample primarily comprised active professionals participating in employer-sponsored health screenings. Importantly, the mean age of the participants was approximately 38 years old. This age group represents a critical life stage frequently burdened with peak occupational demands, intense societal competition, and heavy family responsibilities (26). These compounding socioeconomic stressors significantly elevate their vulnerability to sleep disturbances. Second, the assessment context may have contributed to a higher reporting rate. The questionnaires were administered within a specialized psychological outpatient setting during routine health examinations. This focused clinical environment likely enhanced participants’ health awareness, prompting a more sensitive and forthcoming disclosure of their sleep and psychological symptoms compared to conventional surveys.

Regarding the underlying mechanisms of this association, we observed that FT3, but not TSH or FT4, exerted a significant effect modification on the relationship between psychological symptoms and PSQ. Specifically, the association between psychological symptoms and sleep quality was markedly strengthened in individuals with above-median FT3 levels (PR = 2.26) compared to those with below-median levels (PR = 1.61). While the overall PSQ group exhibited lower mean FT3 levels compared to normal controls (main effect), the association between psychological symptoms and PSQ was stronger in the above-median FT3 group (interaction effect). These two findings can coexist without contradiction, as they reflect different dimensions of the neuroendocrine-sleep relationship: the chronic physiological consequences of sleep deprivation versus neuroendocrine vulnerability. We hypothesize that, on one hand, the overall reduction of FT3 in the PSQ group might reflect a homeostatic down-regulation of the hypothalamic-pituitary-thyroid (HPT) axis in response to chronic sleep fragmentation and prolonged stress, serving as an adaptive mechanism to conserve energy under chronic allostatic load (27). On the other hand, individuals who inherently maintain an above-median FT3 level possess a central nervous system primed in a state of heightened excitability (28, 29). Biologically, FT3 is the primary active form of thyroid hormone. While direct evidence in humans is limited, some experimental models hypothesize that thyroid hormones might indirectly modulate homeostasis within sleep-wake regulatory regions, such as the ventrolateral preoptic nucleus (VLPO) (30, 31). Against this hyperaroused physiological backdrop (32), the convergence of psychological symptoms (e.g., depression and anxiety) easily overwhelms the body’s compensatory capacity for sleep regulation, thereby markedly magnifying the deleterious effects on sleep (33, 34).

These findings provide a novel foundation for precision intervention in health management. In clinical and public health practice, individuals with above-median FT3 levels coupled with elevated psychological symptoms should be recognized as a subpopulation with a disproportionately elevated prevalence of sleep disorders, warranting targeted screening and early intervention. Early implementation of comprehensive interventions-including psychological counseling and circadian rhythm management-is essential not only to improve sleep quality but also to disrupt the vicious cycle of “psychological stress-endocrine dysregulation-sleep impairment (35, 36).

Furthermore, the mixed symptom network analysis identified ‘depression’ as among the most central nodes, while “daytime dysfunction” and “FT3” served as important cross-module bridge nodes. Given that our cohort primarily consisted of active working professionals frequently exposed to high-pressure environments, these findings offer critical clinical insights. Given their high centrality, negative emotions-particularly depression-are highly interconnected with other symptoms in this cohort, suggesting they are prominent features of the comorbidity profile. While our cross-sectional data cannot establish causality, literature suggests that such emotional distress is closely associated with neuroendocrine disruptions, which are in turn associated with FT3 metabolic dysregulation. This, in turn, contributes to reduced daytime energy levels and impaired executive function in both professional and social settings (37). Such persistent daytime fatigue and social withdrawal can intensify emotional exhaustion, decrease daylight activity levels, and disrupt circadian homeostasis, ultimately contributing to nocturnal sleep fragmentation and establishing a self-perpetuating pathogenic feedback loop between psychological symptoms, endocrine dysregulation, and impaired psychosocial functioning (38).

These network characteristics hold significant implications for clinical intervention strategies. Conventional management of sleep disorders often relies on hypnotic medications to prolong nocturnal sleep duration; however, their residual daytime effects may inadvertently aggravate “daytime dysfunction”—a core bridge node in our network—thereby hindering the recovery of neuroendocrine homeostasis (39) Consequently, therapeutic strategies should transcend purely symptom-oriented nocturnal treatments and prioritize the regulation of daytime rhythms and behavioral patterns. Hypothetically, in addition to addressing depressive symptoms, future clinical trials could explore whether integrating evidence-based lifestyle interventions, such as morning outdoor activity or light therapy, can help normalize daytime functioning in this specific subpopulation (40). Enhancing daytime arousal levels and psychosocial executive function may effectively disrupt the solidification of negative emotional states from a behavioral perspective (8). As daytime functioning is restored and psychological exhaustion is mitigated, the resulting improvements are likely to provide feedback-driven benefits for nocturnal sleep quality and metabolic stability—specifically regarding FT3 levels—ultimately achieving synergistic restoration across psychological, physiological, and sleep domains.

This study has several limitations. First, the cross-sectional design precludes definitive causal inferences regarding the temporal sequence among psychological symptoms, thyroid function, and sleep quality (41). Second, there is an inherent temporal mismatch between the SCL-90 (past week) and PSQI (past month), which may introduce temporal bias when assessing chronic stress. Third, relying on a routine health database meant several confounders were unmeasured-including explicit psychotropic prescriptions, shift work, caffeine intake, and geographic factors (e.g., altitude, iodine status)-potentially introducing residual confounding despite our adjustment for self-reported sleep medication. Fourth, network centrality indices represent statistical associations rather than direct mechanisms, requiring future experimental trials for clinical validation. Finally, our cohort of primarily active workers may introduce a “healthy worker effect,” warranting caution when generalizing these findings to more clinically vulnerable psychiatric populations.

## 5 Conclusions

In this health examination population, psychological symptom severity was positively associated with PSQ prevalence, and this association was modified by FT3 levels. In the symptom network, depression and subjective sleep quality were central nodes, while daytime dysfunction and FT3 were key bridge nodes connecting psychological, sleep, and thyroid domains. Longitudinal studies are needed to clarify temporal relationships and to evaluate whether interventions targeting FT3-related pathways and daytime functioning can reduce sleep disorder burden.

## Data Availability

The datasets generated and analyzed during the current study are derived from the internal health management database of Sichuan Provincial People’s Hospital. Due to ethical restrictions and institutional data management policies, the datasets cannot be made publicly available. Eligible researchers may request access to the data for legitimate research purposes by contacting the corresponding author. Requests to access the datasets should be directed to XY, Email: xp_yao@swmu.edu.cn.
