# Supplementary material for: Association of psychological symptoms with poor sleep quality in adults: a cross-sectional network analysis and effect modification by thyroid hormones
Source: Front Endocrinol (Lausanne). 2026 Aug 18;17:1926090. doi: 10.3389/fendo.2026.1926090 (PMC13529465; doi:10.3389/fendo.2026.1926090)
Supplement: Supplementary file 1 [file SupplementaryFile1.docx]

***Supplementary Material***

**Figure S1.** Flowchart of participant selection.

**Figure S2.** Restricted cubic spline (RCS) analysis of the association between free triiodothyronine (FT3) and poor sleep quality.(A) Overall dose-response association between FT3 and poor sleep quality, adjusted for Model 3 covariates (P for nonlinearity = 0.001).(B) Stratified dose-response associations according to psychological symptom severity (GSI below-median vs. above-median groups) (P for nonlinear interaction = 0.194).Solid lines represent predicted prevalence ratios (PR), and shaded areas represent 95% confidence intervals. Reference lines (dashed) are set at PR = 1.0.

**Figure S3.** Stratified correlation heatmaps of psychological symptoms, sleep quality, and thyroid hormones by FT3 median level. (A) Low-FT3 group (n = 1,850); (B) High-FT3 group (n = 1,843).

**Table S1.** Sensitivity Analyses of the Association Between Psychological Symptoms and Poor Sleep Quality

| **Analysis / Variable** | **PR** | **95% CI** | **P** |
| --- | --- | --- | --- |
| **Sensitivity Analysis 1:** Using the PSQI > 5 Cut-off |  |  |  |
| Quartiles of GSI |  |  |  |
| *Q*_1_ | 1.00 | Reference | < 0.001 |
| *Q*_2_ | 1.87 | 1.67~ 2.09 | < 0.001 |
| *Q*_3_ | 2.48 | 2.23~ 2.75 | < 0.001 |
| *Q*_4_ | 2.94 | 2.66~ 3.24 | < 0.001 |
| P for trend | — | — | < 0.001 |
| **Sensitivity Analysis 2:** Excluding Subclinical Thyroid Dysfunction |  |  |  |
| Quartiles of GSI |  |  |  |
| *Q*_1_ | 1.00 | Reference | < 0.001 |
| *Q*_2_ | 2.08 | 1.65~ 2.61 | < 0.001 |
| *Q*_3_ | 3.95 | 3.21~ 4.87 | < 0.001 |
| *Q*_4_ | 5.81 | 4.75~ 7.10 | < 0.001 |
| P for trend | — | — | < 0.001 |

Note：Model adjusted for age, sex, smoking,alcohol drinking, diabetes, hypertension, SBP, TC, BMI, FPG.

**Table S2.** Multicollinearity Diagnostics (Variance Inflation Factors)

| **Variables** | **VIF** | **Variables** | **VIF** |
| --- | --- | --- | --- |
| GSI | 1.05 | Diabetes | 1.63 |
| Age | 1.25 | SBP | 1.85 |
| Sex | 1.57 | TC | 1.15 |
| Smoking | 1.3 | BMI | 1.35 |
| Alcohol drinking | 1.27 | FPG | 1.81 |
| Hypertension | 1.63 |  |  |

**Table S3.** Association Between Individual SCL-90 Dimensions and Poor Sleep Quality

| **Dimension** | **PR (95% CI)** | **P value** |
| --- | --- | --- |
| Somatization | 1.93 (1.83–2.02) | < 0.001 |
| Psychoticism | 1.79 (1.70–1.87) | < 0.001 |
| Obsessive-compulsive | 1.81 (1.74–1.88) | < 0.001 |
| Phobic anxiety | 1.72 (1.63–1.81) | < 0.001 |
| Anxiety | 1.71 (1.64–1.78) | < 0.001 |
| Interpersonal sensitivity | 1.70 (1.63–1.77) | < 0.001 |
| Depression | 1.69 (1.63–1.75) | < 0.001 |
| Paranoid ideation | 1.67 (1.60–1.74) | < 0.001 |
| Hostility | 1.62 (1.56–1.68) | < 0.001 |

Note：Model adjusted for age, sex, smoking,alcohol drinking, diabetes, hypertension, SBP, TC, BMI, FPG.

**Table S4.** Additive Interaction Measures for the Association Between Psychological Symptoms and FT3 Levels on Poor Sleep Quality

| **Thyroid hormone** | **Stratum/Analysis** | **PR (95% CI)/ Estimate** |
| --- | --- | --- |
| FT3 | Additive Interaction Measures: |  |
|  | — RERI | -0.28 (-0.48 to -0.08) |
|  | —AP | -0.21 (-0.35 to -0.06) |
|  | —SI | 0.57 (0.38 to 0.76) |

Note：All models were adjusted for age, smoking, alcohol drinking, hypertension, diabetes,SBP, BMI, and FPG (consistent with Model 3).

**Table S5.** Sex-Stratified and Euthyroid Sensitivity Analyses

| **Analysis Category** | **Subgroup / Model Specification** | **PR (95% CI)** | **P value / P for Interaction*** |
| --- | --- | --- | --- |
| **1. Sex-Stratified Analysis** |  |  |  |
| TSH Model | Male | 1.94 (1.82–2.07) | — |
|  | Female | 1.89 (1.76–2.03) | 0.375* |
| FT3 Model | Male | 1.76 (1.65–1.87) | — |
|  | Female | 1.75 (1.62–1.89) | 0.983* |
| FT4 Model | Male | 1.93 (1.80–2.06) | — |
|  | Female | 1.91 (1.78–2.05) | 0.304* |
| **2. Sensitivity Analysis** |  |  |  |
| Euthyroid Cohort | Continuous GSI × TSH | 0.95 (0.89–1.01) | 0.099 |
|  | Continuous GSI × FT3 | 1.44 (1.31–1.57) | < 0.001 |
|  | Continuous GSI × FT4 | 1.02 (1.00–1.05) | 0.079 |

Note：All models were adjusted for age, smoking, alcohol drinking, hypertension, diabetes, SBP, BMI, and FPG (consistent with Model 3).* P values for interaction in sex-stratified analysis represent the three-way interaction term (Psychological symptoms×Thyroid hormone×Sex). Euthyroid sensitivity analysis restricted the cohort to participants with all thyroid hormone levels within normal laboratory reference ranges (n = 3,019).

**Table S6.** Centrality and Bridge Centrality Metrics of the Mixed Symptom Network

| **Community**  **(Module)** | **Node** | **Variable (Symptom/Index)** | **Expected Influence**  **(EI)** | **Bridge Expected Influence** |
| --- | --- | --- | --- | --- |
| Psychological | SCL90-1 | Somatization | 0.962 | 0.366 |
|  | SCL90-2 | Obsessive-compulsive | 1.035 | 0.212 |
|  | SCL90-3 | Interpersonal sensitivity | 1.147 | 0 |
|  | SCL90-4 | Depression | 1.115 | 0.16 |
|  | SCL90-5 | Anxiety | 1.205 | 0.045 |
|  | SCL90-6 | Hostility | 0.682 | 0.014 |
|  | SCL90-7 | Phobic anxiety | 0.627 | 0.095 |
|  | SCL90-8 | Paranoid ideation | 0.845 | 0.025 |
|  | SCL90-9 | Psychoticism | 1.19 | 0.078 |
| Sleep | PSQI1 | Subjective sleep quality | 1.077 | 0.052 |
|  | PSQI2 | Sleep latency | 0.72 | 0.154 |
|  | PSQI3 | Sleep duration | 0.615 | 0.104 |
|  | PSQI4 | Habitual sleep efficiency | 0.572 | 0.028 |
|  | PSQI5 | Sleep disturbances | 0.596 | 0.252 |
|  | PSQI6 | Use of sleeping medications | 0.13 | 0.14 |
|  | PSQI7 | Daytime dysfunction | 0.611 | 0.474 |
| Thyroid | FT3 | Free triiodothyronine | 0.088 | 0.374 |
|  | FT4 | Free thyroxine | 0.252 | 0.067 |
|  | TSH | Thyroid-stimulating hormone | -0.056 | 0.118 |
